# The Protective Role of Alpha-Ketoglutaric Acid on the Growth and Bone Development of Experimentally Induced Perinatal Growth-Retarded Piglets

**DOI:** 10.3390/ani11010137

**Published:** 2021-01-10

**Authors:** Ewa Tomaszewska, Natalia Burmańczuk, Piotr Dobrowolski, Małgorzata Świątkiewicz, Janine Donaldson, Artur Burmańczuk, Maria Mielnik-Błaszczak, Damian Kuc, Szymon Milewski, Siemowit Muszyński

**Affiliations:** 1 Department of Animal Physiology, Faculty of Veterinary Medicine, University of Life Sciences in Lublin, Akademicka St. 12, 20-950 Lublin, Poland; jgajski@wp.pl; 2 Department of Functional Anatomy and Cytobiology, Faculty of Biology and Biotechnology, Maria Curie-Sklodowska University, Akademicka St. 19, 20-033 Lublin, Poland; piotr.dobrowolski@umcs.lublin.pl; 3 Department of Animal Nutrition and Feed Science, National Research Institute of Animal Production, Krakowska St. 1, 32-083 Balice, Poland; malgorzata.swiatkiewicz@izoo.krakow.pl; 4 Faculty of Health Sciences, School of Physiology, University of the Witwatersrand, 7 York Road, Parktown, Johannesburg 2193, South Africa; janine.donaldson@wits.ac.za; 5 Faculty of Veterinary Medicine, Institute of Preclinical Veterinary Sciences, University of Life Sciences in Lublin, Akademicka St. 12, 20-950 Lublin, Poland; artur.burmanczuk@up.lublin.pl; 6 Department of Developmental Dentistry, Medical University of Lublin, 7 Karmelicka St., 20-081 Lublin, Poland; maria.mielnik-blaszczak@umlub.pl (M.M.-B.); damiankuc@vp.pl (D.K.); 7 Department of Biophysics, Faculty of Environmental Biology, University of Life Sciences in Lublin, Akademicka St. 13, 20-950 Lublin, Poland; xszymon47@gmail.com (S.M.); siemowit.muszynski@up.lublin.pl (S.M.)

**Keywords:** α-ketoglutaric acid, pigs, glucocorticoid, perinatal growth-retardation, bone

## Abstract

**Simple Summary:**

Perinatal growth restriction is a significant health issue that predisposes to a reduced rate of postnatal weight gain and the development of numerous diseases later in life. In livestock production, growth restricted animals require more time to reach slaughter weight. In this study, we examined the effects of long-term administration of alpha-ketoglutaric acid (AKG) on the growth and development of experimentally-induced, perinatal growth-retarded piglets.

**Abstract:**

The effect of alpha-ketoglutaric acid (AKG) supplementation to experimentally-induced, perinatal growth-retarded piglets was examined. Sows were treated with a synthetic glucocorticoid (Gc) during the last 25 days of pregnancy, and after the birth, piglets were randomly divided into three groups depending on the treatment. The Gc/Gc + AKG and Gc/AKG groups born by Gc-treated sows after the birth were treated with Gc or Gc + AKG for 35 days. Significantly lower serum growth hormone, IGF-I, osteocalcin, leptin, and cortisol concentrations were observed in the Gc/Gc + AKG group, while the bone alkaline phosphatase activity was significantly higher. Serum insulin concentration was higher in the control group. Serum alanine, lysine, histidine, and tryptophan concentrations were higher in the Gc/Gc + AKG and Gc/AKG groups. The perinatal action of Gc significantly affects histomorphometry of articular cartilage and trabecular bone and bone mechanics. The results clearly showed that dietary AKG had positive effects with regards to the profile of free amino acids. Taking into account the function of AKG as an energy donor and stimulator of collagen synthesis, it can be concluded that the anabolic role of AKG may be the main mechanism responsible for its protective effect against the GC-induced perinatal intensified catabolic state.

## 1. Introduction

To our knowledge, most studies regarding livestock growth and development are predominantly focused on postnatal development. However, recently more and more research is being undertaken to study prenatal and even perinatal development and growth retardation. The results of these studies show that the structural and functional development of organs, as well as the achievement of full efficiency with regards to metabolic processes within the organism as a whole and within individual organs, is determined genetically and is influenced by many factors. Particular attention is paid to nutritional and hormonal factors and their role in the maximum use of the potential genetic abilities of a given species during the postnatal developmental period. Many studies show that both the prenatal and perinatal periods play an important role in the possible development of disturbances in postnatal development [1,2,3,4,5,6,7,8,9,10,11,12]. Studies highlighting the negative effects of perinatal programming, which inhibit the postnatal development of an organism, may allow us to take appropriate preventive actions.

Glucocorticoids (Gc) play an important role in the proper prenatal development of a fetus and are involved in the functional maturation of vital organs and systems (e.g., skeletal, respiratory, and gastrointestinal systems) [13,14,15,16,17,18,19,20]. Moreover, immediately before and after delivery, the most important role in the adaptation of the fetus to the new environment is played by the hormones of the adrenal cortex, under the control of which are the lungs, liver, intestines, bone marrow, nervous system, and even the placenta [21,22,23,24]. The adrenal cortex hormones influence the developmental processes, including the development of adaptive mechanisms in the neonate that provide them with a certain level of functional suitability, necessary to ensure life under difficult neonatal conditions compared to the fetal period. The endocrine action of Gc is evident in the changes in function of two axes: the hypothalamic-pituitary-adrenal gland axis (HPA axis) and the growth hormone-insulin-insulin-like growth factor I axis (somatotropin axis) [2,25,26,27,28,29,30].

Endogenous Gc are known to be released in stressed pregnant females [31]. The same effect could be achieved by synthetic Gc treatment of pregnant females [32,33]. Newborns are also under considerable stress. They have to compete with siblings for food, and they have to withstand temperature variations, pathogens, parasites, bad ventilation, lack of comfort, lack of enough space or sleep, and bad conditions during transport and handling. Studies have shown that excess Gc inhibits fetal growth and reduces placental size, depending on the duration of exposure [31,34]. Several animal models have been used to study experimentally-induced intrauterine growth restriction (IUGR). Some of them have shown that prenatal Gc exposure transiently or permanently inhibited longitudinal bone growth, and the effect is gender-dependent [10,33,35].

The longitudinal bone growth and the maturity of the skeletal system determine locomotive functions ensuring the proper movement and behavior of animals. The skeletal system as a place for the attachment of muscles not only provides support and maintains mineral homeostasis but determines the general welfare of pigs and economy in fattening [36,37,38].

Many studies have shown that glutamine and its derivatives, such as glutamate, can counteract growth inhibition resulting from a negative nitrogen balance in the body as a result of stress, malnutrition, and a poorly balanced diet [39,40,41,42,43,44,45]. Glutamine is a conditionally essential amino acid found mainly in skeletal muscle cells and accounts for more than half of the total pool of free amino acids in the body; it is the main amino acid in the cerebrospinal fluid [39]. In the case of glutamine deficiency, glutamine can be produced from other amino acids, causing quantitative and qualitative losses in the pool of amino acids necessary primarily for muscles. Alpha-ketoglutaric acid (AKG; 2-oxoketoglutaric acid, 2-Ox), the precursor of glutamine, is a key intermediate in many metabolic pathways, including the Krebs cycle [40,43,46,47,48]. Alpha-ketoglutaric acid is also a precursor of proline, asparagine, and arginine, which are necessary for the synthesis of collagen proteins [46,47]. Nearly 90% of free glutamine, or the salt of glutamic acid, is used by the cells of the gastrointestinal tract as the main source of energy in oxidative processes, where it is metabolized to carbon dioxide and has been found to stimulate the growth of intestinal cells, accelerating their proliferation and differentiation [44,49,50]. Mucins, which provide the molecular framework for the mucus present in the gastrointestinal tract and are important in the maintenance of the intestinal barrier, are composed of hexosamines, for the synthesis of which glutamine is used [40]. There is also a correlation between the level of glutamine in the gastrointestinal tract, skeletal muscles, and blood serum and the Gc administered. Dog studies have shown that Gc increase the metabolism of glutamine in the gastrointestinal tract [44]. Dietary supplementation with free glutamine, under conditions of excess Gc, increases the activity of glutaminase and increases the level of glutathione in the jejunum. In addition, a higher glutamine content has been noted in the blood serum, skeletal muscle and intestinal mucosa of rats subjected to catabolic stress [49,51].

There are a few studies evaluating both the short-term and long-term effects of AKG treatment on bone metabolism in experimentally-induced IUGR animal models [7,10,33,52,53,54]. However, there is a dearth of information on the effect of AKG treatment on bone metabolism in perinatally growth-retarded piglets.

Therefore, the current experiment was designed to evaluate whether AKG given during postnatal life accelerates growth and bone development in experimentally Gc-induced, perinatal, growth-retarded piglets. Taking into account the beneficial effects of AKG administration in the presence of excess Gc during the prenatal or neonatal period, we decided to verify the hypothesis that AKG administration could be useful to eliminate or attenuate the side effects of Gc in piglets subjected to chronic Gc exposure during the perinatal period or during the prenatal period only.

The study was carried out to evaluate the effects of neonatal oral administration of AKG, on general growth as well as bone mineral density, mechanical properties, bone trabecular histomorphometry, as well as growth plate cartilage structure in experimentally-induced (maternal or perinatal Gc exposure) growth-retarded neonatal piglets. For this purpose, the present study involved multiple methods, including blood serum free amino acids and basal biochemical analysis in relation to general growth; while the assessment of bone quality included mechanical testing, dual X-ray absorptiometry, quantitative computer tomography, and histological preparation.

## 2. Materials and Methods

### 2.1. Ethical Approval

All experimental procedures were approved by The Local Ethics Committee on Animal Experimentation of University of Life Sciences in Lublin (11/05), Poland, and were complied with the Directive 2010/63/EU of the European Parliament and of the Council on the protection of animals used for scientific purposes. Throughout the whole experiment, the health status of pregnant sows and piglets was regularly monitored by a veterinarian. Except for suckling behavior, other behavior was not monitored.

### 2.2. Pregnant Sows

**Figure 1:**
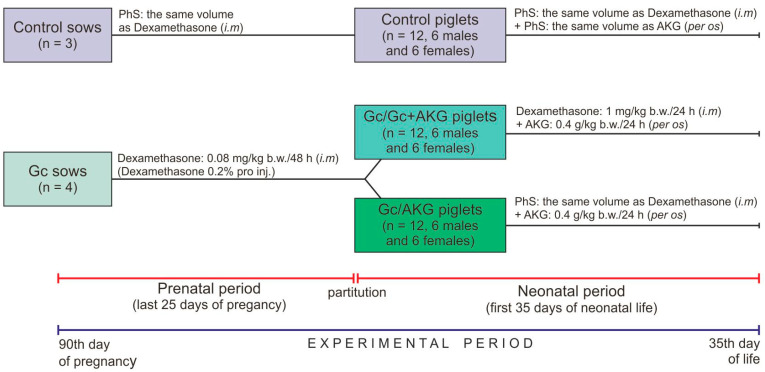
Scheme showing the experimental design. From the 90th day of gestation to parturition dexamethasone was administered to n = 4 sows belonging to Gc group at the dose 0.08 mg /kg b.w. every second day. After delivery, 24 newborns of Gc-treated sows were randomly assigned to one of the two groups: the Gc/Gc + AKG group receiving dexamethasone (1 mg/kg b.w./24 h *i.m*) and supplemented with alpha-ketoglutaric acid (AKG) (0.4 g/kg b.w./24 h *per os*) or the Gc/AKG group supplemented with AKG (0.4 g/kg b.w./24 h *per os*) only, compromising of 6 males and 6 females each. Twelve piglets delivered by untreated control sows (n = 3) belonged to the control group. The experiment lasted until the 35th day of piglets’ neonatal life.

Seven, clinically healthy, multiparous (second parity) sows, of the Large White Polish breed, were sired by the same boar (Large White Polish breed) and singly housed in separate cages that provide the adequate living conditions and under standard rearing conditions (controlled temperature and humidity, 12:12-h light-dark cycle). The sows had free access to fresh water and were fed twice a day with a properly balanced commercial feed mixture for pregnant sows, supplied in equal doses for all sows (3.0 kg/day/sow; Table 1). All diets were formulated to meet or exceed the requirement with regards to nutrients, metabolizable energy and mineral elements, during pregnancy and lactation [55]. During lactation, the feeding rate was dependent on the litter size to cover their nutritional requirements. Sows were randomly divided into two groups: control (n = 3) and Gc-treated (n = 4). The experimental period lasted during high pregnancy and lactation. Glucocorticoid (dexamethasone, Dexamethasone 0.2%, Eurovet Animal Health B.V., Bladel, The Netherlands) was administered from the 90th day of pregnancy by intramuscular injection, in the morning (0.08 mg/kg body weight/every second day), to the parturition; the control sows received only physiological saline (PhS, 0.9% NaCl) in the same volume (Figure 1). The dose and period of Gc treatment was determined from previous studies, which have also shown that dexamethasone administration did not influence gestation length, or the mean number of stillborn and live born piglets in litters delivered from Gc-treated sows, compared to sows not treated with dexamethasone [6,8,33,56].

### 2.3. Piglets

Newborn piglets of both sexes, from sows in the control and experimental groups, were weighed immediately after their birth and before colostrum collection. Piglets with the body weight closest to the litter average were randomly divided into three groups (n = 12 in each group, six gilts and six boars), depending on the treatment. Piglets from the control group of sows belonged to the control group, while piglets from the Gc-treated sows were divided in two groups (Gc/Gc + AKG and Gc/AKG) (Figure 1). Piglets in the Gc/Gc + AKG group were injected with Gc (1 mg/kg b.w./daily) and supplemented with 0.4 g/kg b.w./day of AKG (α-Ketoglutaric acid, #75890, Sigma-Aldrich, St. Louis, MO, USA) up to 35 days of age, while those from the Gc/AKG group were only supplemented with the same dose of AKG during the neonatal period, also up to 35 days of age. Piglets in the control group received PhS both intramuscularly (the same route of administration as Gc) and per os (the same route of administration as AKG) in corresponding volumes. The Ethics Committee, in accordance to “the 3Rs” principle of avoiding the unnecessary use of experimental animals, recommended excluding from the experiment the AKG supplemented or Gc-treated piglets born by Gc-untreated sows due to the fact that many other studies using this experimental scheme have already been performed [5,6,35,57].

The piglets were weighed every day to determine the doses of AKG and dexamethasone required (0.4 g/kg b.w. as a solution). On the 35th day of neonatal life, blood samples were collected from each piglet and allowed to clot in blood collection tubes, and then centrifuged (3000× *g* for 15 min at 4 °C) to obtain serum. The piglets were then weighed and euthanized by an intravenous injection of pentobarbital (Morbital, Biowet, Puławy, Poland). The experiment period was comprised of a total of 60 days: a gestational period of 25 days (from day 90 to parturition) and then the first 35 days of neonatal life of the piglets.

### 2.4. Blood Serum Analysis

Serum total cholesterol, triacylglycerols (TG), low-density lipoproteins (LDL), high-density lipoproteins (HDL), urea, creatinine, uric acid, total protein, glucose, albumin, alanine transaminase (ALT), aspartate transaminase (AST), and alkaline phosphatase (ALP) was assessed using an automatic biochemistry analyzer (Mindray BS-120, Bio-Medical Electronics, Shenzhen, China). Commercial ready-to-use test kits were purchased from Alfa Diagnostics (Warsaw, Poland). All analysis procedures were verified with the use of multiparametric control serum (BioCal, Alfa Diagnostics, Warsaw, Poland).

Serum concentration of selected hormones and the activity of bone alkaline phosphatase (BAP) were determined using commercial porcine-specific enzyme-linked immunosorbent assay (ELISA) kits: growth hormone (GH; Diagnostic System Laboratories, Inc., Webster, TX, USA), leptin (Diagnostic System Laboratories, Inc., Webster, TX, USA), osteocalcin (Diagnostic System Laboratories, Inc., Webster, TX, USA), insulin (Mercodia, Uppsala, Sweden), cortisol (Uscn Life Science Inc., Wuhan, China), insulin-like growth factor I (IGF-I; Uscn Life Science Inc., Wuhan, China), bone alkaline phosphatase (BAP; Quidel Corporation, San Diego, CA, USA). All procedures were performed according to the manufacturer’s protocols. Samples were analyzed in duplicate using a Benchmark Plus microplate spectrophotometer (Bio-Rad Laboratories, Inc., Hercules, CA, USA). Results were calculated using standard curves created in individual tests.

Determination of free amino acid concentration in serum was performed with the use of ion-exchange chromatography and an INGOS AAA-400 apparatus for the automatic analysis of amino acids (INGOS Corp., Prague, Czech Republic). Amino acids were separated using an OSTION LG FA analytic column (3 mm × 200 mm). Lithium citrate buffers were used for amino acid separation. Amino acids were derivatized with ninhydrin and their determination was performed on the basis of retention time in comparison to the standards, using photocell combined with a computer. The apparatus-integrated MIKRO software (INGOS Corp., Prague, Czech Republic) was used for amino acid evaluation.

The concentration of macro- and micro-elements (Na, Mg, P, S, Ca, Fe, Cu, and Zn) in lyophilized serum samples was determined using an energy dispersive X-ray fluorescence (EDXRF) spectrometry method (ED 2000 spectrometer, Oxford Instruments, Buckinghamshire, UK) [58,59]. The serum concentration of determined elements was expresses as percentage (Na, S), mg/g (Mg, P, Ca) or µg/g (Fe, Cu, Zn) of dry matter of the lyophilized sample.

### 2.5. Bone Analysis

Immediately after euthanasia, the femora from individual pigs were dissected, cleaned from adherent tissues, wrapped in gauze soaked in isotonic saline, and frozen below −40 °C until further analyses. In subsequent stages of analyses, left femora were used for determination of bone weight, length, densitometry, and mechanical testing, while right femora were earmarked for bone midshaft geometry measurements and histomorphometric analysis.

The measurement of bone mineral density (BMD) and bone mineral content (BMC) for the whole bone was performed using a dual-energy X-ray absorptiometry (DXA) method on an XR 43 X-ray densitometer (Norland, Fort Atkinson, WI, USA), calibrated before measurements with bone phantoms of known BMD [60]. Volumetric bone mineral density was measured by quantitative computed tomography using a LightSpeed VCT apparatus (GE Healthcare, Pollards Wood, UK). Compact bone mineral density (BMDc) was assessed at the midpoint of the bone diaphysis. Trabecular bone mineral density (BMDt) was measured in the distal metaphysis with the scan positioned just below the calcification zone of the growth plate [61].

Assessment of the mechanical properties of the bones was carried out using a three-point bending Zwick Z010 testing system (Zwick-Roell GmbH & Co., Ulm, Germany). The bones were positioned on two supports (distance span 33 mm, equal to 40% of the mean bone length) and a constant, perpendicular force was exerted by the moving crosshead (10 mm/min) on the midshaft of the bones until fracture. The ultimate strength (F max) and yield load (F yield) were determined from registered load-displacement curves [62].

After the measurements, femora were cut across in the midpoint of the bone diaphysis with a diamond bandsaw (MBS 240/E, Proxxon GmbH, Foehren, Germany) and the cross-sectional geometric properties of the bone diaphysis (cross-sectional area, CSA; mean relative wall thickness, MRWT; and cortical index, CI) were determined on the basis of measurements of bone cross-sectional diameters (internal and external), measured with a digital caliper [63].

### 2.6. Bone Histomorphometry

Cylindrical, 20 mm thick samples of the lateral condyle were cut off with a diamond bandsaw, perpendicularly to the articular surface from the same anatomical position from the middle of the of distal epiphysis. Samples were fixed in phosphate-buffered 4% paraformaldehyde for 24 h at room temperature, decalcified in EDTA solution (10%, pH 7.4), dehydrated through a graded ethanol series, and embedded in paraffin. Sections were cut with a microtome at a thickness of 4 μm and mounted on the SuperFrost Plus slides (Thermo Scientific, Germany) and processed for routine staining procedures. Slides were stained with Goldner’s trichrome, in order to evaluate trabecular bone histomorphometry and basal morphology of the growth plate cartilage [62]. Stained slides were observed using a light microscope (CX43, Olympus, Tokyo, Japan).

To assess the morphology of the trabecular bone at the epiphysis and metaphysis, the relative bone volume (BV/TV), trabecular thickness (Tb.Th), trabecular separation (Tb.Sp), trabecular number (Tb.N), and specific bone surface (BS/BV) were calculated from the microscopic images of the bone epiphysis and metaphysis, using ImageJ software [64].

For the growth plate cartilage, four zones (reserve, proliferation, hypertrophy, and calcification) were identified and their thickness was determined. All measurements were performed at eight, a priori sites along the growth plate cartilage and an average was reported [8,56,65]. Analysis of the images collected was performed with the use of CellSens software (Olympus, Tokyo, Japan).

### 2.7. Statistical Analysis

Data were analyzed using Statistica 13.3 (TIBCO Software Inc., Palo Alto, CA, USA). The normality of data distribution was tested using the Shapiro–Wilk test. A one-way ANOVA was used to assess parametric data with treatment as the fixed effect and piglet as the experimental unit. Treatment means were compared using Tukey’s HSD (honest significant difference) test. For all tests, a criterion α level of *p* ≤ 0.05 was used to determine statistical significance. All data are expressed as means and SD (standard deviation).

## 3. Results

### 3.1. Body Weight

Piglets born from Gc-treated sows (1298 ± 165 g) weighed significantly less compared to those from the control sows (1476 ± 132 g; *p* = 0.030). At 35 days of age, piglets in the control group weighed 6755 ± 775 g, whilst piglets in the Gc/Gc + AKG group had a significantly lowest body weight (4326 ± 708 g), with the piglets in the Gc/AKG group being the heaviest (8375 ± 759 g; *p* < 0.001).

### 3.2. Serum Biochemical and Hormonal Analysis

Blood serum concentrations of the selected hormones analyzed in 35-day-old piglets are presented in Table 2. Significantly lower serum GH and IGF-I concentrations were observed in piglets in the Gc/Gc + AKG group compared to those in the Gc/AKG and control groups. Serum leptin concentrations were the lowest in piglets in the Gc/Gc + AKG group compared to the other groups, while the highest leptin concentration was observed in the control group. The BAP activity was significantly higher in the piglets in the Gc/Gc + AKG and Gc/AKG groups compared to that of those in the control group. Serum osteocalcin concentration was the highest in piglets in the Gc/AKG group, with it being lower in the control group, and the lowest in piglets in the Gc/Gc + AKG group. Serum insulin concentration was higher in the piglets in the control group compared to those in the Gc/Gc + AKG and Gc/AKG groups. Serum cortisol concentration was the lowest in piglets in the Gc/Gc + AKG group, higher in the Gc/AKG group, and the highest in piglets in the control group.

Serum concentrations of micro- and macro elements are presented in Table 3. Serum Cu and Zn concentrations were lower in piglets in the Gc/Gc + AKG group compared to the other groups. The serum concentrations of the other elements assessed were similar in all three groups.

The serum concentrations of selected amino acids are presented in Table 4. Serum asparagines, glycine, and glutamic acid concentrations were increased in the pigs in the Gc/Gc + AKG and Gc/AKG groups compared to the control group. Serum glutamine and serine concentrations were highest in the piglets in the Gc/Gc + AKG group, lower in the Gc/AKG group, and the lowest in the control group. Serum proline and leucine concentrations were higher in the piglets in the Gc/AKG group compared to those in the other groups. Serum alanine concentrations were higher in the piglets in the Gc/Gc + AKG group compared to those in the control group. Serum lysine, histidine, and tryptophan concentrations were higher in the piglets in the Gc/Gc + AKG and Gc/AKG groups compared to those in the control group. Methionine was the highest in the Gc/AKG group compared to the control group where was the lowest. Valine was the highest in the Gc/AKG group compared to the lowest concentration noted in the control group. No other differences in serum amino acid concentrations between groups were observed.

Basal biochemical serum parameters are presented in Table 5. Higher serum total cholesterol, LDL, and creatinine concentrations were observed in the piglets in the Gc/Gc + AKG group compared to those in the other groups. Serum HDL concentration was increased in piglets in the Gc/Gc + AKG group compared to those in the control group. Lower serum glucose and urea concentrations and a higher serum uric acid concentration were noted in piglets in the Gc/Gc + AKG group compared to that observed in the other groups. Higher serum ALT, AST, and ALP activity was observed in the piglets in the Gc/AKG group compared to those in the control and Gc/Gc + AKG groups. Serum total protein concentration was lower in the piglets in the Gc/Gc + AKG group compared to those in the other groups. No other differences in serum biochemical parameters between groups were observed.

### 3.3. Bone Analysis

Femur weight was the lowest in the piglets in the Gc/Gc + AKG group, with it being higher in the control group, and the highest in the Gc/AKG group (Table 6). Femur length was lower in the piglets in the Gc/Gc + AKG group compared to those in the other groups. The lowest BMD was also noted in the piglets in the Gc/Gc + AKG group, with it being higher in the control group and the highest in the piglets in the Gc/AKG group. The lowest BMC was observed in the piglets in the Gc/Gc + AKG group. BMC was higher in the control group and the highest Gc/AKG group. Computer tomography analysis showed the lowest compact and trabecular bone density in the piglets in the Gc/Gc + AKG group, with it being higher in the control group, and the highest in the Gc/AKG group. Ultimate strength and yield load, as well as cross-sectional area of the bones, were the lowest in the piglets in the Gc/Gc + AKG group, higher in the control group, and the highest in piglets in the Gc/AKG group. MRWT was higher in the piglets in the Gc/AKG group compared to those in the other groups. Cortical index was significantly higher in the Gc/AKG group compared only to the Gc/Gc + AKG group.

Histomorphometrical parameters of trabecular bone in the epiphysis and metaphysis are presented in Table 7. In the epiphysis, relative bone volume (BV/TV) and trabecular thickness were the lowest in piglets from the Gc/Gc + AKG group, higher in the Gc/AKG group and the highest in piglets in the control group. The highest trabecular space was observed in piglets in the Gc/Gc + AKG group, with it being lower in the Gc/AKG group and the lowest in piglets in the control group. Trabecular number in the epiphysis was lower in the piglets in the Gc/Gc + AKG group compared to those in the control group. The specific bone surface (BS/BV) was the lowest in piglets in the Gc/Gc + AKG group, higher in the Gc/AKG group, and the highest in piglets in the control group.

In the metaphysis, relative bone volume (BV/TV) was lower in the piglets in the Gc/Gc + AKG and Gc/AKG groups compared to those in the control group. Trabecular thickness was the lowest in piglets in the Gc/Gc + AKG group, higher in the Gc/AKG group and the highest in piglets in the control group. Higher trabecular space was observed in piglets in the Gc/Gc + AKG and Gc/AKG groups, compared to those in the control group. Trabecular number in the metaphysis was not significantly different between groups. The specific bone surface (BS/BV) in the piglets in the Gc/Gc + AKG group was lower compared to that of piglets in the other groups, where BS/BV was similar.

Structural information obtained from the analysis of the femoral growth plate cartilage is presented in Table 8. A thinner reserve zone thickness and thicker proliferative zone were observed in piglets in the Gc/Gc + AKG group compared to that of piglets in the other groups. The thickness of the calcification zone was the lowest in the piglets in the Gc/Gc + AKG group compared to those in the control group.

## 4. Discussion

Previous studies have shown that Gc administered to pregnant sows has a dual metabolic effect on prenatal and neonatal growth and development of offspring born from these sows. This effect depends mainly on Gc dose and the time of Gc administration during pregnancy. The administration of 0.03 mg/kg b.w. of dexamethasone to pregnant sows, from the 91st day of gestation, increased serum glucose concentrations, resulting in macrosomia of newborns and an increase in long bone mass [6,35]. The opposite effect was obtained when pregnant sows were treated with 0.08 mg/kg b.w. of dexamethasone during the same period of pregnancy. Newborn piglets in this case were significantly smaller compared to those in the control group [34]. The effects of dexamethasone administration at a dose of 0.03 mg/kg b.w. to sows, during the last 45 days of pregnancy (from day 70), were found to be dependent on the sex of the offspring. The body weight of female newborns was not affected by dexamethasone treatment, while newborn boars were found to be significantly smaller than their counterparts in the control group. These differences are still visible during the weaning period and even into adulthood [7,10,33].

The data obtained in the presented study showed that the fetal exposure to Gc excess from the 90th day of pregnancy in sows until the end of the pregnancy, significantly inhibited prenatal development and led to decreased term body weight of newborn offspring. The differences between the current results and previous reports concerning term body weight are probably due to variations in the dose of Gc used, as well as the time of administration during gestation [66].

The results obtained from the current study regarding the body weight of piglets at weaning showed further inhibition of growth in piglets exposed to perinatal Gc, despite AKG supplementation. The general catabolic effects of dexamethasone during the neonatal period has been confirmed in previous piglet studies [49,50,67]. Weiler et al. [12] induced a catabolic effect in piglets by the administration of dexamethasone for 15 days, from 7 days of age, at a dose of 0.5 mg/kg b.w./day. In contrast, low doses of Gc have been shown to increase body weight [68]. Similar effects were observed by Michel and Cabanac (1999), where a low dose of dexamethasone in the drinking water of rats (0.025–0.05 µg/mL) increased body weight, and the opposite effect was observed in the high-dose group (0.25–3.1 µg/mL) [69]. Caroll [70] and Seaman-Bridges et al. [67] investigated the effects of dexamethasone administration in the early neonatal period on the development and growth of piglets until the slaughter weight. Caroll [70] observed a 12.2% higher body weight in 18-day-old piglets administered with a single dose of dexamethasone (1.0 mg/kg b.w.) one hour after birth. The other study showed, that the difference in growth rate between the dexamethasone-treated piglets and the control piglets persisted until the slaughter period (at 25 weeks of age), where pigs exposed to dexamethasone in the first hour of life were about 5 kg heavier compared to controls [67]. The opposite effect was observed in the current study, where piglets were treated with dexamethasone only during the prenatal period (the Gc/AKG group). However, this anabolic and anti-catabolic effect of AKG is consistent with the results of numerous previous studies. The protective properties of AKG (glutamine precursor), as a nutritional supplement, against the many negative effects of dexamethasone have been demonstrated in numerous previous experiments [5,10,29,56,61,71,72,73,74,75,76,77,78]. Nevertheless, such contrasting results with regards to the action of AKG, as those observed in our piglets, perinatally treated with Gc, have never been noted before. It should also be considered that the metabolic variation that Gc treatment exerts on body weight is dependent on Gc dose and the type of steroid administered as well as the duration of therapy. The same variation of natural endogenous Gc effect can be observed in breeding when stimulus factor acts [79].

Changes in the development of piglet long bones were also observed in the current study. The changes in bone mass and length observed in the perinatally Gc-treated group were consistent with the changes observed in body weight. A visible anabolic effect of AKG treatment was observed with regards to the bone mass of the piglets in the prenatally Gc-treated group, where piglets were not only heavier but also had heavier bones. Densitometric analysis (BMD and BMC) showed that postnatal exposure to excess Gc negatively affected bone mineralization, especially that of the piglets in the Gc/Gc + AKG group. This result proved that disturbances in general metabolic and growth processes resulted in changes in the skeletal mineralization process, irrespective of AKG supplementation. At the same time, QCT analysis showed a greater decrease in density of the compacted bone compared to that observed in the trabecular bone. These changes can be attributed to the Gc overload during the prenatal period, which resulted in very low term body weights, with further Gc exposure during the neonatal period intensifying the catabolic effect, irrespective of AKG supplementation. Other studies have also shown that dexamethasone administered to sows during the last 24 days of pregnancy, even when administered at low doses (0.03 mg/kg b.w./48 h), inhibits fetuses or newborn offspring bone mineralization and growth [35,57]. However, when neonatal animals were additionally treated with AKG, a stronger anti-osteoporotic effect could be expected. This anabolic effect of AKG on bone mineralization was only observed in the piglets that were exposed to Gc during the prenatal period in the current study (Gc/AKG group), and the QCT analysis confirmed a greater intensity of changes in mineralization in the compacted bone. The bone densitometric results obtained in the present study were confirmed by the results obtained from the mechanical testing. The growth inhibition and decreases in the bone mechanical parameters assessed were also reflected in the bone geometric measurements. Histomorphometric analysis of the trabecular bone of the femur showed advanced trabecular bone loss (reduction of trabecular thickness and an increase in intra-trabecular space). This means that perinatal Gc excess disrupted the process of bone formation, although growth plate structure was not drastically affected. The opposite effect was noted in piglets that were treated with Gc only during the prenatal period, where AKG supplementation resulted in increased ultimate strength and yield load, as well as better bone mineralization.

However, many previous studies have shown that AKG does not always play a protective role against the catabolic action of Gc. Postnatal dexamethasone treatment (1.0 mg/kg b.w./24 h), along with AKG supplementation from birth up to weaning in piglets, has been shown to result in decreased body weight, bone length, and bone mass, as well as decreased bone mechanical endurance, mineralization, and cortical index. Moreover, the bone histomorphometric and histological analyses confirmed the loss of trabeculae [61]. Dexamethasone administered during the neonatal period also reduces the concentration of growth hormone, IGF-I, osteocalcin, cortisol, and urea, without any effect on alkaline phosphatase. There is also an increase in the concentration of glycine, glutamine, and glutamate and a decrease in serum proline, which additionally may indicate a decrease in collagen synthesis, showing that the loss of bone tissue may not only be related to a decrease in bone mineralization [61]. Another study involving prenatal Gc treatment also observed a decrease not only in the weight of the long bones, but also in their mechanical endurance and bone mineralization [56]. The Gc treatment was found to affect the growth plate of the bones and resulted in increased bone alkaline phosphatase. The increased bone alkaline phosphatase is associated with intensified bone tissue resorption. However, no changes in serum growth hormone and osteocalcin were observed [56]. The effects of Gc treatment of pregnant sows are long term and have been observed in the offspring of the Gc-treated sows at 9 months of age [10,33]. Over and above the reduction in body weight, bone mass, and bone length, a reduction in bone mechanical parameters and a reduced degree of bone mineralization, with reduced histomorphometry of trabecular bone are still observed. The decreases in serum osteocalcin and IGF-I concentration are also still observed in the offspring. Thus, in these 9-month-old piglets, osteopenic bone is present [10]. Postnatal AKG supplementation, following Gc exposure, has been shown to have both anti-catabolic and anti-osteoporotic effects [10,33,56]. On one hand, postnatal AKG supplementation has been shown to almost completely prevent the negative effects of prenatally administered Gc [10]. However, on the other hand, a study on developing, 7-week-old rats treated with orally-administered AKG for a further 7 weeks showed that AKG supplementation resulted in a higher body weight and longer femurs, but did not affect bone weight [80]. Although the AKG supplementation in these rats also resulted in a thicker growth plate, higher bone collagen content, and thinner trabeculae [80].

Bone and cartilage homeostasis is regulated by a multifactorial system that includes growth hormone, IGF-1, leptin, and OPG. IGF-I plays a dominant role in the development of the fetus [81]. Leptin is closely related to serum IGF-I concentration, but independently regulates fetal growth. It is assumed that at the beginning of life, leptin can directly stimulate the growth of bones in length and thickness, through angiogenesis and stimulation of connective tissue synthesis as well as by influencing overall metabolism [82]. Moreover, leptin can directly stimulate bone formation or inhibit bone resorption via the RANKL/RANK/OPG system, i.e., the essential signaling pathway through which osteoblasts regulate the number of active osteoclasts and thus bone resorption [11]. The prevailing view is that leptin can alter the OPG-to-RANKL ratio in the bone microenvironment in favor of OPG, leading to a reduction in the active osteoclast pool and bone resorption by inhibiting RANKL expression and/or stimulating OPG expression in osteoblasts [83,84]. It is therefore possible that in the present experiment, the administration of AKG to piglets (the Gc/AKG group) regulated the levels of leptin and IGF-I, and in addition, growth hormone improved cell proliferation and maturation, and thus bone development was observed. The increase in bone alkaline phosphatase observed in both of our experimental groups could indicate intensified bone tissue remodeling as well. As a result, the intensification of bone turnover processes leads to bone loss, mainly in the group exposed to the further catabolic effects of Gc (regardless of the level of total alkaline phosphatase). However, there was the lack of differences in serum calcium and phosphorus concentrations indicating that in animals the physiological mechanisms maintaining homeostasis within the organism have been maintained. Furthermore, the current study showed changes mainly in the content of copper and zinc; elements that primarily affect the process of collagen synthesis, the main organic element of bone tissue and cartilage.

Additional changes after the AKG supplementation were observed in our piglets in serum glucose concentrations. Bone resorption is not only a part of the continuous remodeling process of the skeleton, but also stimulates insulin release into the bloodstream and glucose uptake by cells. First, insulin acts on the osteoblasts, which in turn activate the osteoclasts, resulting in the destruction of the bone tissue. When bone tissue pH drops as a result of this process, osteocalcin is activated and insulin release is stimulated. Consequently, disruption of skeletal metabolic processes can induce diabetes, and some bone-strengthening drugs used to treat osteoporosis can affect glucose levels [85,86].

In the current study, a decreased serum cortisol concentration was observed in piglets from the Gc/Gc + AKG group, which is a result of the Gc-induced suppression of the adrenal glands, which in turn led to bone decalcification and weakness. Theories about the effect of Gc therapy on the hypothalamic-pituitary-adrenal (HPA) axis vary. Some authors argue that Gc used in recommended doses do not cause clinically significant disturbances in HPA axis function. Others have shown that adrenal suppression occurs in less than 18% of patients treated with low doses of Gc, 26.5% of patients treated with medium doses, and in 36.3% of patients treated with high doses of Gc [87,88].

Considering the negative effects of Gc, especially during the prenatal period, when sensitivity of the organism to metabolic and hormonal factors is very high, it is very important to define its role in prenatal and perinatal programming of postnatal growth and metabolism. Prenatal exposure to Gc excess is associated with changes in metabolic processes in the liver, resulting in excessive lipid accumulation and an increase in blood glucose and cholesterol concentration [89,90,91]. As shown in a previous study, AKG given neonatally to piglets can prevent liver damage after prenatal exposure to Gc [5]. Śliwa et al. [5] have also shown that AKG administered at a dose of 0.4 mg/kg b.w. to piglets born from sows receiving dexamethasone at a dose of 0.03 mg/kg b.w. during the last 45 days of pregnancy, lowers cholesterol concentration. In the present study, we also observed a protective role of AKG against Gc-induced metabolic changes; however, this was only in the Gc/AKG group, even though increased total cholesterol and LDL concentrations were observed in perinatally Gc-treated piglets (Gc/Gc + AKG). The Gc/Gc + AKG piglets also had increased serum creatine concentrations, which did not result from increased muscle mass, since these piglets were the smallest of the three groups. However, Gc overload could increase the breakdown of protein and creatinine and thus increase serum creatinine as a product of creatine phosphate breakdown from muscle protein metabolism. The increased serum creatinine observed in the Gc/Gc + AKG piglets is consistent with the results of an earlier study where increased serum creatinine was also observed in piglets treated with dexamethasone (1.0 mg/kg b.w. daily), together with AKG, at the same doses as those used in our study [80].

In the current study, besides the increase in serum creatinine observed, changes in serum ALT and AST were also noted. Alterations in ALT and AST activity are indicative not only of disturbances in liver function due to Gc excess, but also of muscle breakdown [92]. An increase in transaminase activity may be one of the first symptoms of a disturbance in muscle metabolism, but typically AST activity is close to or exceeds ALT activity, as was observed in both groups of our Gc overloaded piglets. On the other hand, as mentioned, ALT is a liver function marker along with other biochemical parameters, such as alkaline phosphatase (ALP). ALP activity increased in the piglets in the Gc/AKG group, where significant changes in liver function markers were noted. Why the liver was more metabolically overloaded in this group should be further investigated.

It should be noted that AKG is a precursor of glutamine and glutamate. Glutamine is synthesized in living organisms from glutamic acid and does not need to be present in food. It constitutes over 60% of all muscle-building amino acids, which are its largest reservoir. Under unfavorable conditions (stress, illness, exercise), the need for glutamine (an important energy source for many cells) increases rapidly, which thus reduces its supply. The body produces small amounts of this amino acid, but under conditions where there is a large deficit, glutamine is obtained from other sources, such as skeletal muscles as a result of catabolic reactions. The administration of AKG prevents this glutamine loss from muscles [5,61,77,78]. Analysis of the profile of endogenous and exogenous amino acids in the blood serum of our piglets showed a significant increase in the concentration of glutamic acid and glutamine in both experimental groups. These are amino acids formed from AKG, and they belong to the so-called glutamate family, which includes glutamate and its derivatives: glutamine, proline and arginine. The increase in serum concentration of glutamic acid and glutamine observed in our piglets likely resulted in an increase in the level of asparagine. Asparagine is synthesized in the body from oxaloacetic acid, as a result of the transfer of two amino groups, in turn from glutamate and glutamine, with the participation of the enzymes transaminase and aspartate synthetase. Furthermore, among all the amino acids of the asparagine family (asparagine, methionine, lysine, and isoleucine), besides asparagine, we also observed an increased lysine concentration in the Gc/AKG and Gc/Gc + AKG groups. Lysine participates in proteinogenesis and histone modification [93] and is an important feed additive due to its key role in optimizing the growth of pigs for the production of meat [94].

One of the hallmarks of proliferating cells is their dependency on aerobic glycolysis, which is known as the Warburg effect [95]. The enhanced uptake of glucose sustains energy production and the synthesis of macromolecular precursors. In addition to glucose dependency, mammalian cells also rely on extracellular glutamine to support cell survival and growth [96]. The glutamine-derived AKG is an essential component of glutamine-dependent cell survival. Moreover, in some conditions, glutamine-dependent asparagine synthesis from aspartate is observed. The only known use of asparagine in mammalian cells is in protein synthesis. Asparagine is the regulator of a pathway that induces translation-dependent apoptosis [96].

Furthermore, the concentration of glycine was also changed in the current study. Glycine can also be produced from glutamate by glutamate aminotransferase, or from alanine by alanine aminotransferase, the concentrations of which were increased in our piglets. There was also a significant increase in serum serine concentrations, which in turn is produced from glycine. Free serine is identified in a wide variety of mammalian tissues and cells at relatively high concentrations. Serine is a neuromodulator that binds to the glycine-binding site of the N-methyl-d-Asp (NMDA) receptor, a subtype of the l-glutamate (l-Glu) receptor and potentiates glutamatergic neurotransmission in the central nervous system [97]. Alanine, which can be synthesized from pyruvate and branched chain amino acids such as valine, leucine, and isoleucine, was increased in Gc/Gc + AKG piglets. Changes in serum concentrations of alanine precursor valine, were also observed in our study. Perhaps the increase in serum alanine observed in our perinatally Gc-treated piglets supplemented with AKG resulted in lowered concentrations of glucose. In mammals, alanine plays a key role in the glucose–alanine cycle between tissues and liver. Alanine is involved in metabolic pathways such as glycolysis, gluconeogenesis, and the citric acid cycle. The glucose–alanine cycle allows for the removal of pyruvate and glutamate from muscle and ensures transport to the liver, where pyruvate is used to regenerate glucose and the glucose returns to the muscles as an energy source.

Therefore, the concentrations of many free amino acids were elevated in AKG-treated piglets in order to maintain growth and prevent the degradation of proteins and growth retardation, for example, an increase in serum leucine concentration was noted in the Gc/AKG piglets.

However, it must be noted that changes in the serum amino acids profile could result not only from the AKG supplementation but also from the mutual relationships of their metabolic pathways or catabolic action of Gc. For example, Gc are known to increase bone turnover and collagen degradation, which results in the increase in serum proline. Proline is the most abundant amino acid in sow colostrum and milk. After suckling, proline increases fourfold in the plasma and liver to become the most abundant amino acid in these tissues. A high proline concentration in neonatal piglet tissue is needed for the synthesis of connective tissue and bone matrix [98]. Collagen represents about 30% of total protein in young pigs [99]. Many of the analyzed amino acids (glutamine, glutamate, leucine, and proline) modulate gene expression and enhance growth of the small intestine and skeletal muscle [100]. Tryptophan, an aromatic amino acid, was increased after AKG supplementation. Tryptophan is a biochemical precursor of serotonin and melatonin; it is also involved in modulation of the endocrine system (cortisol, as well as growth hormone) [101].

The results of the current study on the analysis of the profile of selected amino acids confirms the results of an earlier experiment carried out on piglets, which showed an increase in serine, glutamine, glutamate, glycine, arginine, and methionine after prenatal dexamethasone treatment, although at different doses (3.0 mg/kg b.w./48 h) and time (from 70th day of pregnancy) [5]. Another study, also carried out on piglets treated with dexamethasone during the neonatal period, at the same dose as that used during the neonatal part of the current study (1.0 mg/kg b.w./ 24 h), showed that the administration of Gc resulted in an increase in the concentration of glycine, glutamine, and glutamate. Moreover, in the group receiving AKG after dexamethasone treatment, only proline concentration was increased [61]. The results obtained are consistent with other studies and confirm the possibility of prenatal programming as well as neonatal or perinatal modification through functional nutrition.

## 5. Conclusions

The results obtained in the present study clearly show that dietary AKG supplementation can significantly reduce the side effects of Gc exposure, dependent mainly on their metabolic effects and on the time during which the Gc excess occurs. It might seem as if AKG was not protective against the strong catabolic effects triggered by perinatal Gc exposure; however, even though a decrease in body weight was observed, AKG still had positive effects with regards to the profile of amino acids in Gc-treated piglets. This is possibly due to the many different metabolic pathways AKG is involved in. Taking into account the function of AKG as an indirect energy donor and stimulator of collagen synthesis, it can be concluded that the anabolic role of AKG may be the main mechanism responsible for its protective effect against the GC-induced intensified catabolic state.

## Figures and Tables

**Table 1:** Composition of feed mixture used for sows during pregnancy and lactation.

Ingredients	Content, %
Wheat	19.00
Barley	23.30
Corn	20.00
Soybean meal	17.00
Wheat bran	8.00
Dried alfalfa	6.00
Rapeseed oil	4.00
Calcium carbonate	1.00
Calcium phosphate	0.60
Salt	0.35
Vitamin-mineral premix	0.50
L-Lysine	0.20
DL-Methionine	0.028
L-Threonine	0.03

**Table 2:** The concentration of selected hormones and the activity of bone alkaline phosphatase (BAP) in the blood serum of 35-day-old piglets.

Item	Control	Gc/Gc + AKG	Gc/AKG	*p*-Value
GH, ng/mL	5.00 ± 1.02 ^b^	1.28 ± 0.25 ^a^	4.54 ± 0.84 ^b^	<0.001
IGF-I, ng/mL	76.1 ± 10.8 ^b^	31.5 ± 8.3 ^a^	70.6 ± 12.2 ^b^	<0.001
Leptin, ng/mL	3.31 ± 0.45 ^c^	0.80 ± 0.33 ^a^	1.43 ± 0.62 ^b^	<0.001
BAP, U/L	89.3 ± 9.6 ^a^	205 ± 16 ^b^	200 ± 12 ^b^	<0.001
Osteocalcin, ng/mL	37.4 ± 3.5 ^b^	29.5 ± 1.7 ^a^	45.0 ± 3.1 ^c^	<0.001
Insulin, μg/mL	0.096 ± 0.016 ^b^	0.057 ± 0.026 ^a^	0.064 ± 0.020 ^a^	<0.001
Cortisol, ng/mL	53.7 ± 9.2 ^c^	17.8 ± 6.9 ^a^	44.0 ± 8.8 ^b^	<0.001

Data are mean values ± SD (n = 12 in each group). GH—growth hormone; IGF-1—insulin-like growth factor 1; BAP—bone alkaline phosphatase. a, b, c—values in rows with different letters differ significantly (*p* ≤ 0.05; Tukey’s HSD test).

**Table 3:** The concentration of micro- and macro-elements in the blood serum of 35-day-old piglets.

Item	Control	Gc/Gc + AKG	Gc/AKG	*p*-Value
Na, %	4.99 ± 0.34	4.88 ± 0.44	5.20 ± 0.66	0.292
Mg, mg/g	4.53 ± 1.11	3.30 ± 0.59	3.79 ± 0.95	0.069
P, mg/g	2.86 ± 0.27	2.59 ± 0.33	2.61 ± 0.28	0.057
S, %	1.61 ± 0.04	1.64 ± 0.05	1.63 ± 0.07	0.403
Ca, mg/g	1.21 ± 0.12	1.28 ± 0.11	1.23 ± 0.13	0.352
Fe, μg/g	39.3 ± 9.6	33.7 ± 4.8	41.0 ± 6.9	0.053
Cu, μg/g	6.47 ± 0.47 ^b^	5.29 ± 0.78 ^a^	6.22 ± 0.46 ^b^	<0.001
Zn, μg/g	7.45 ± 1.03 ^b^	3.57 ± 1.17 ^a^	6.33 ± 1.51 ^b^	<0.001

Data are mean values ± SD (n = 12 in each group). a, b—values in rows with different letters differ significantly (*p* ≤ 0.05; Tukey’s HSD test).

**Table 4:** Free amino acid concentrations (nmol/mL) in the blood serum of 35-day-old piglets.

Item	Control	Gc/Gc + AKG	Gc/AKG	*p*-Value
Alanine	562 ± 59 ^a^	699 ± 50 ^b^	631 ± 102 ^a,b^	<0.001
Arginine	121 ± 31	114 ± 17	122 ± 23	0.684
31 Asparagine	10.8 ± 3.2 ^a^	16.4 ± 3.1 ^b^	17.7 ± 2.0 ^b^	<0.001
Citrulline	50.0 ± 19.5	65.8 ± 7.7	52.1 ± 11.5	0.017
Glutamic acid	97 ± 40 ^a^	167 ± 18 ^b^	176 ± 34 ^b^	<0.001
Glutamine	169 ± 42 ^a^	364 ± 27 ^c^	298 ± 67 ^b^	<0.001
Glycine	524 ± 78 ^a^	769 ± 35 ^b^	684 ± 94 ^b^	<0.001
Histidine	78.3 ± 11.4 ^a^	97.5 ± 10.3 ^b^	98.3 ± 18.8 ^b^	0.002
Isoleucine	78.3 ± 16.9	87.9 ± 12.4	86.9 ± 15.8	0.248
Leucine	140 ± 28 ^a^	200 ± 19 ^b^	180 ± 21 ^a,b^	<0.001
Lysine	184 ± 25 ^a^	242 ± 19 ^b^	284 ± 42 ^b^	<0.001
Methionine	24.4 ± 9.1 ^a^	35.6 ± 5.8 ^b^	30.7 ± 7.7 ^a,b^	0.004
Proline	693 ± 138 ^a^	615 ± 148 ^a^	1303 ± 302 ^b^	<0.001
Serine	93 ± 18 ^a^	165 ± 11 ^c^	129 ± 27 ^b^	<0.001
Tryptophan	33.2 ± 6.2 ^a^	34.4 ± 2.0 ^a^	51.0 ± 6.8 ^b^	<0.001
Valine	194 ± 29 ^a^	205 ± 42 ^a,b^	243 ± 44 ^b^	0.011

Data are mean values ± SD (n = 12 in each group). a, b, c—values in rows with different letters differ significantly (*p* ≤ 0.05; Tukey’s HSD test).

**Table 5:** Basal biochemical parameters in the blood serum of 35-day-old piglets.

Item	Control	Gc/Gc + AKG	Gc/AKG	*p*-Value
Total cholesterol, mg/dL	61.0 ± 13.9 ^a^	74.8 ± 8.6 ^b^	51.6 ± 10.9 ^a^	<0.001
TG, mg/dL	19.0 ± 2.4	21.2 ± 3.8	21.3 ± 6.3	0.373
HDL, mg/dL	3.79 ± 0.60 ^a^	4.54 ± 0.26 ^b^	4.20 ± 0.58 ^a,b^	0.004
LDL, mg/dL	48.2 ± 10.2 ^a^	65.5 ± 5.2 ^b^	44.1 ± 8.5 ^a^	<0.001
Urea, mg/dL	26.8 ± 5.0 ^b^	17.6 ± 3.0 ^a^	26.3 ± 5.8 ^b^	<0.001
Creatinine, mg/dL	1.40 ± 0.19 ^a^	1.90 ± 0.28 ^b^	1.70 ± 0.16 ^a^	<0.001
Uric acid, mg/dL	0.727 ± 0.360 ^a^	1.212 ± 0.47 ^b^	0.739 ± 0.499 ^a^	0.017
Total protein, g/L	74.9 ± 2.9 ^b^	65.4 ± 3.7 ^a^	71.9 ± 3.04 ^b^	<0.001
Albumin, g/dL	3.33 ± 0.19	3.54 ± 0.27	3.50 ± 0.31	0.129
Glucose, mg	118 ± 17 ^b^	84 ± 20.0 ^a^	133 ± 14 ^b^	<0.001
AST, U/L	28.1 ± 5.4 ^a^	32.3 ± 5.9 ^a^	54.0 ± 7.3 ^b^	<0.001
ALT, U/L	26.7 ± 5.2 ^a^	26.3 ± 4.8 ^a^	40.2 ± 5.4 ^b^	<0.001
ALP, U/L	208 ± 42 ^a^	230 ± 37 ^a^	292 ± 24 ^b^	<0.001

Data are mean values ± SD (n = 12 in each group). TG—triacylglycerol; HDL—high-density lipoprotein; LDL—low-density lipoprotein; AST—aspartate transaminase; ALT—alanine transaminase; ALP—alkaline phosphatase. a, b—values in rows with different letters differ significantly (*p* ≤ 0.05; Tukey’s HSD test).

**Table 6:** Morphology, geometry, density, and mechanical properties of the femur of 35-day-old piglets.

Item	Control	Gc/Gc + AKG	Gc/AKG	*p*-Value
Weight, g	31.9 ± 2.6 ^b^	21.5 ± 3.3 ^a^	40.1 ± 3.5 ^c^	<0.001
Length, cm	8.61 ± 0.36 ^b^	7.46 ± 0.35 ^a^	8.91 ± 0.29 ^b^	<0.001
CSA, mm^2^	54.8 ± 6.2 ^b^	38.3 ± 4.0 ^a^	72.6 ± 5.0 ^c^	<0.001
MRWT	0.587 ± 0.095 ^a^	0.577 ± 0.057 ^a^	0.674 ± 0.083 ^b^	0.010
CI, %	36.7 ± 3.8 ^a,b^	36.4 ± 2.4 ^a^	39.7 ± 2.9 ^b^	0.024
BMD, g/cm^2^	0.363 ± 0.017 ^b^	0.252 ± 0.019 ^a^	0.421 ± 0.015 ^c^	<0.001
BMC, g	2.73 ± 0.32 ^b^	1.47 ± 0.34 ^a^	3.60 ± 0.26 ^c^	<0.001
BMDc, g/cm^3^	2.13 ± 0.03 ^b^	2.00 ± 0.06 ^a^	2.30 ± 0.06 ^c^	<0.001
BMDt, g/cm^3^	1.34 ± 0.03 ^b^	1.28 ± 0.04 ^a^	1.37 ± 0.01 ^c^	<0.001
F max, N	1350 ± 248 ^b^	702 ± 162 ^a^	1958 ± 249 ^c^	<0.001
F yield, N	814 ± 112 ^b^	578 ± 117 ^a^	1563 ± 141 ^c^	<0.001

Data are mean values ± SD (n = 12 in each group). CSA—cross-sectional area; MRWT—mean relative wall thickness; CI—cortical index; BMD—bone mineral density; BMC—bone mineral content; BMDc—compact bone mineral density of central part of bone mid-diaphysis; BMDt—trabecular bone mineral density below the calcification zone of the growth plate; F max—ultimate strength; F yield—yield load. a, b, c—values in rows with different letters differ significantly (*p* ≤ 0.05; Tukey’s HSD test).

**Table 7:** Histomorphometry measurements of trabeculea in epiphysis and metaphysis of the femur of 35-day-old piglets.

Item	Control	Gc/Gc + AKG	Gc/AKG	*p*-Value
*Epiphysis*				
BV/TV, %	18.7 ± 0.8 ^c^	9.9 ± 0.8 ^a^	12.9 ± 0.8 ^b^	<0.001
Tb.Th, μm	32.4 ± 1.5 ^c^	19.9 ± 0.9 ^a^	24.6 ± 2.4 ^b^	<0.001
Tb.Sp, μm	233 ± 6 ^a^	285 ± 6 ^c^	262 ± 12 ^b^	<0.001
Tb.N, /mm	5.79 ± 0.39 ^b^	4.98 ± 0.47 ^a^	5.32 ± 0.54 ^a,b^	<0.001
BS/BV, %	6.52 ± 0.29 ^c^	3.95 ± 0.26 ^a^	4.89 ± 0.45 ^b^	<0.001
*Metaphysis*				
BV/TV, %	17.6 ± 1.0 ^b^	11.4 ± 0.9 ^a^	12.4 ± 1.3 ^a^	<0.001
Tb.Th, μm	30.1 ± 0.9 ^c^	20.1 ± 1.8 ^a^	27.1 ± 2.4 ^b^	<0.001
Tb.Sp, μm	214 ± 12 ^a^	259 ± 30 ^b^	241 ± 11 ^b^	<0.001
Tb.N, /mm	5.85 ± 0.34	5.23 ± 0.82	5.26 ± 0.75	0.052
BS/BV, %	6.0 ± 0.45 ^b^	3.99 ± 0.35 ^a^	5.46 ± 0.61 ^b^	<0.001

Data are mean values ± SD (n = 12 in each group). BV/TV—relative bone volume; Tb.Th—trabecular thickness; BS/BV—specific bone surface; Tb.Sp.—trabecular space; Tb.N.—trabecular number. a, b, c—values in rows with different letters differ significantly (*p* ≤ 0.05; Tukey’s HSD test).

**Table 8:** The structure of the femoral growth plate cartilage in 35-day-old piglets.

Item	Control	Gc/Gc + AKG	Gc/AKG	*p*-Value
Reserve zone, μm	382 ± 46 ^b^	287 ± 34 ^a^	374 ± 45 ^b^	<0.001
Proliferative zone, μm	133 ± 27 ^a^	243 ± 40 ^b^	141 ± 45 ^a^	<0.001
Hypertrophy zone, μm	108 ± 26	105 ± 22	96 ± 16	0.382
Calcification zone, μm	163 ± 42 ^b^	115 ± 18 ^a^	131 ± 29 ^a,b^	0.002

Data are mean values ± SD (n = 12 in each group). a, b—values in rows with different letters differ significantly (*p* ≤ 0.05; Tukey’s HSD test).

## Data Availability

The data presented in this study are available on request from the corresponding author.
